# Molecular evidence of SARS-CoV-2 detection on cockroaches in Shiraz, southwestern Iran and potential implications for mechanical carriage

**DOI:** 10.1016/j.parepi.2026.e00558

**Published:** 2026-10-23

**Authors:** Maryam Motevasel, Mozafar Vahedi, Marzeyeh Jamalidost, Mohsen Kalantari

**Affiliations:** a Department of Medical Laboratory Sciences, School of Paramedical Sciences, Shiraz University of Medical Sciences, Shiraz, Iran; b Student Research Committee, Department of Medical Entomology and Vector Control, School of Health, Shiraz University of Medical Sciences, Shiraz, Iran; c Clinical Microbiology Research Center, Shiraz University of Medical Sciences, Shiraz, Iran; d Research Center for Health Sciences, Institute of Health, Department of Medical Entomology and Vector Control, School of Health, Shiraz University of Medical Sciences, Shiraz, Iran

**Keywords:** Cockroach, SARS-CoV-2, Mechanical transmission, RT-PCR, Shiraz, Iran

## Abstract

Cockroaches are known vectors of various pathogens due to their unsanitary feeding habits and close association with human habitats. The COVID-19 pandemic has raised concerns about potential non-human transmission routes, including mechanical transmission by insects. This study investigates whether cockroaches can carry SARS-CoV-2 RNA and their potential role in mechanical transmission in Shiraz, Iran. Using real-time RT-PCR (positivity threshold: Ct < 38.0 for both RdRp and N genes), we molecularly examined 200 cockroaches (100 *Blattella germanica* and 100 *Periplaneta americana*) randomly selected from a total of 400 collected specimens. An additional 200 specimens were allocated to insectarium-based vertical transmission experiments. SARS-CoV-2 RNA was detected on external surfaces of 59/200 specimens (29.5%; 95% CI: 23.4–36.3%), with significantly higher detection in *P. americana* from hospital sewage (35/50, 70%; 95% CI: 55.9–81.2%) than *B. germanica* from dormitory kitchens (24/50, 48%; 95% CI: 34.2–62.2%; χ^2^ = 5.01, *p* = 0.025). Internal viral RNA was detected in only 11/200 specimens (5.5%; 95% CI: 3.1–9.6%), with significantly lower loads than surface samples (mean surface-to-internal ratio: 15.2:1; *p* < 0.001). No viral RNA was detected in 200 nymphs or 50 egg capsules from insectarium-reared populations, indicating no evidence of transovarial transmission under experimental conditions. These findings demonstrate that cockroaches can carry SARS-CoV-2 RNA on their external surfaces, suggesting potential for mechanical carriage rather than biological transmission. However, as RT-PCR detects nucleic acids rather than infectious virus, our results do not establish mechanical transmission. Further research incorporating viral viability assays is needed to determine whether cockroaches can transmit infectious SARS-CoV-2 and contribute to COVID-19 epidemiology.

## Highlights

• This study investigates whether cockroaches can carry SARS-CoV-2 RNA on their surfaces, but does not establish infectious transmission.
• SARS-CoV-2 RNA was detected on 29.5% of molecularly analyzed cockroach surfaces, with hospital sewage showing the highest prevalence (70%).
• No viral RNA was detected in nymphs or egg capsules, despite surface contamination.
• Detection of SARS-CoV-2 RNA on cockroach surfaces suggests potential for mechanical carrier though transmission remains unconfirmed.
• RT-PCR was used to detect SARS-CoV-2 RNA on cockroach surfaces and tissues, offering a robust molecular assessment.

## 1 Introduction

Cockroaches (order Blattodea) are among the most resilient and ancient insects, having existed for over 300 million years (Brenner and Kramer, 2019). They are ubiquitous in human environments, particularly in tropical and subtropical regions, where they thrive in unsanitary conditions (Abudin et al., 2023). Cockroaches are known to harbor and transmit a wide range of pathogens, including bacteria, viruses, and parasites, making them significant vectors of disease (Donkor, 2020). Their feeding habits, which include consuming feces, decaying organic matter, and human food, coupled with their tendency to regurgitate and defecate on food sources, contribute to their role in the spread of infectious diseases (Haile et al., 2018).

Insects are implicated in the transmission of bacterial diseases such as salmonellosis, cholera, Wolbachia, and dysentery, as well as viral diseases like poliomyelitis (Alipour et al., 2021; Crespo et al., 2025). The medical importance of insects such as cockroaches has been well-documented. Additionally, cockroaches can carry parasitic worm eggs and cause allergic reactions in humans, including asthma and dermatitis. Their ability to mechanically transmit pathogens by contaminating surfaces and food sources has been a subject of extensive research (Geng et al., 2025).

The emergence of SARS-CoV-2, the virus responsible for the COVID-19 pandemic, has prompted investigations into potential non-human transmission routes (Gryseels et al., 2021). While the primary mode of SARS-CoV-2 transmission is through respiratory droplets and close human contact, the virus has been detected on various surfaces, raising concerns about fomite transmission (Jo et al., 2021). Studies have shown that SARS-CoV-2 can remain viable on surfaces for hours to days, depending on environmental conditions such as temperature and humidity (Kampf et al., 2020). This environmental persistence has led to speculation about the potential role of insects—particularly cockroaches, which frequent contaminated surfaces—in the mechanical carriage of the virus (Dehghani and Kassiri, 2020; Ismail et al., 2020).

Cockroaches are known to frequent environments where SARS-CoV-2 contamination is likely, such as hospitals, dormitories, and food preparation areas (Cabeça et al., 2013; Corman et al., 2018; Chauhan et al., 2020). Their ability to carry pathogens on their external surfaces and in their digestive tracts, combined with their synanthropic behavior, makes them plausible candidates for mechanical carriage of viral pathogens (Donkor, 2020). Furthermore, the virus's presence in fecal matter, which cockroaches may come into contact with, adds another layer of complexity to their potential role in transmission (Davari et al., 2023; Wu et al., 2020b).

While previous studies in Iran have reported molecular detection of SARS-CoV-2 in cockroaches, this study extends the investigation to novel dimensions (Fotouhi-Ardakani et al., 2021; Kalantari et al., 2023). Unlike prior studies limited to hospitals, we include dormitories/educational institutions, assess vertical transmission via nymphs/egg capsules, and compare viral loads across species *(B. germanica* vs. *P. americana*). This approach addresses gaps in understanding mechanical versus biological transmission roles.

This study aims to investigate whether cockroaches can carry SARS-CoV-2 RNA on their external surfaces and internal tissues, and to explore the potential for mechanical carrier of the virus in Shiraz, Iran. Importantly, we distinguish between detection of viral RNA and the capacity for infectious transmission, which would require additional viability studies beyond the scope of this molecular investigation. By examining the external surfaces and internal tissues of cockroaches collected from various locations, we seek to determine whether these insects can harbor the virus and potentially facilitate its spread. Additionally, we explore the possibility of vertical and horizontal transmission of the virus through cockroach nymphs and egg capsules. Our findings contribute to a broader understanding of the potential non-human transmission routes of SARS-CoV-2 and inform public health strategies to mitigate the spread of COVID-19.

## 2 Materials and methods

### 2.1 Study area

This cross-sectional study was conducted in Shiraz, the capital of Fars province in southern Iran. The city has a semi-tropical climate with an average annual temperature of 18 °C and relative humidity of 41%. Cockroaches were collected from various locations, including Ali Asghar Hospital (a primary COVID-19 treatment center), the campus of the Paramedical School and School of Public Health at Shiraz University of Medical Sciences, and Shahid Dastgheib dormitory, where medical students reside.

### 2.2 Sample size calculation

The minimum sample size (*n* = 378) was calculated a priori based on an expected SARS-CoV-2 detection rate of 5% (conservative estimate from prior studies of pathogen carriage by cockroaches) with a 95% confidence level and 5% margin of error. A total of 400 cockroaches were collected to ensure robustness, accounting for potential exclusions during analysis. Of these, 200 specimens were randomly selected for molecular detection of SARS-CoV-2 RNA using RT-PCR. All prevalence estimates are based on this molecularly analyzed subset (*n* = 200), as the remaining 200 specimens were not tested for SARS-CoV-2 RNA but were instead allocated exclusively to the insectarium-based vertical transmission experiments. This sample size (*n* = 200) provides 80% power to detect a 5% prevalence with 95% confidence (exact binomial confidence intervals: 2.4% to 9.1%). The remaining 200 specimens were allocated exclusively to the insectarium-based vertical transmission experiments, where they were reared to produce nymphs (*n* = 200) and egg capsules (*n* = 50) for subsequent molecular testing. This dual allocation was necessary to investigate both environmental contamination and the potential for transovarial transmission, as the latter required viable breeding populations. It is important to note that prevalence estimates (e.g., 59/200, 29.5%) are based on the 200 specimens molecularly analyzed, not the total 400 collected. This approach aligns with methodologies used in similar vector competence studies (Fotouhi-Ardakani et al., 2021).

### 2.3 Ethical approval

This study was conducted in accordance with international, national, and institutional ethical guidelines. All experimental protocols were approved by the Science and Ethics Committee of Shiraz University of Medical Sciences (Approved ID: IR.SUMS.SCHEANUT.REC.1400.855).

### 2.4 Cockroach collection and identification

Cockroaches were collected over a two-month period in 2022 by trained entomologists. American cockroaches (*Periplaneta americana*) were primarily collected from sewage systems, while German cockroaches (*Blattella germanica*) were collected from indoor environments such as kitchens and rooms. A minimum of 378 samples were required for molecular analysis based on similar studies. Collected cockroaches were individually placed in sterile glass vials and transported to the laboratory for morphological identification and molecular analysis (Fig. 1).

**Fig. 1:**
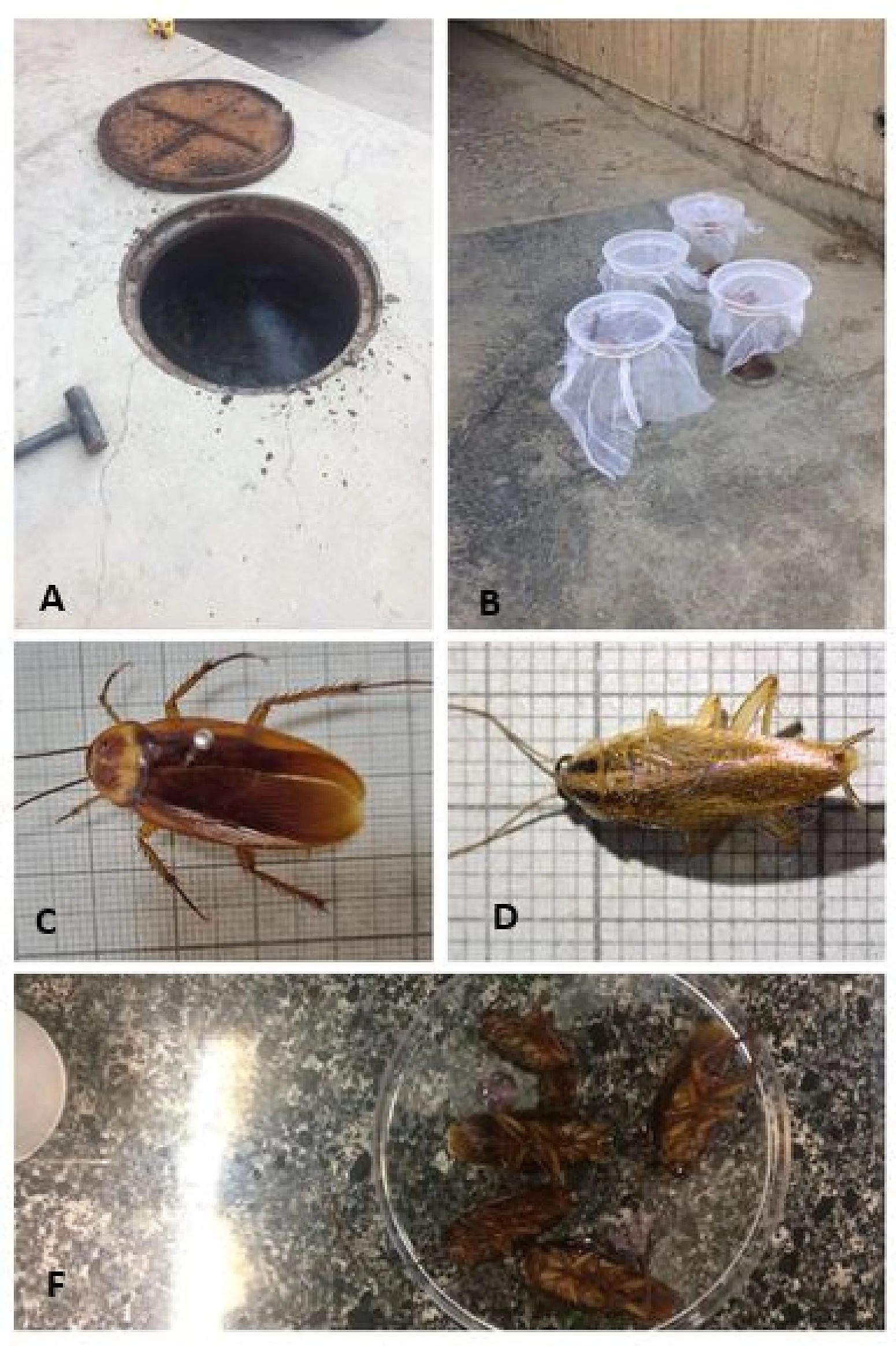
Cockroaches Collection (A, B), Identification (C, D), and Preparation (F) for molecular analysis.

A subset of 200 specimens was randomly subjected to molecular analysis to detect the presence of SARS-CoV-2 RNA using real-time RT-PCR targeting conserved regions of the viral genome. All prevalence estimates reported in this study are based on this 200-specimen molecular analysis cohort.

To investigate the possibility of vertical transmission—specifically transovarial transmission—of SARS-CoV-2, adult cockroaches were reared in an insectarium under controlled environmental conditions. A total of 200 adult cockroaches (100 *B. germanica* and 100 *P. americana*) were selected for rearing, comprising all specimens that were not allocated to the molecular detection cohort (as described in Section 2.2). Importantly, to prevent cross-contamination and to distinguish between vertical transmission and environmental re-exposure, the reared adults were strictly segregated into two separate insectarium units based on their initial RT-PCR status:

**Group A (Positive Adults):** 59 adults that had tested positive for SARS-CoV-2 RNA on external surfaces (35 *P. americana* from Ali Asghar Hospital and 24 *B. germanica* from Shahid Dastgheib Dormitory).

**Group B (Negative Adults):** 141 adults that had tested negative for SARS-CoV-2 RNA on external surfaces (65 *P. americana* and 76 *B. germanica* from all locations).

This segregation ensured that any viral RNA detected in offspring could be attributed to transovarial transmission from virus-exposed parents rather than to environmental contamination from co-rearing with positive specimens. The insectarium was maintained at a constant temperature of 27 ± 1 °C, relative humidity of 65 ± 5%, and a 12:12 h light-dark (L:D) photoperiod. Cockroaches were housed in ventilated plastic containers provided with shelters to facilitate natural behavior and oviposition. Sterile distilled water was supplied ad libitum via a cotton-plugged glass petri dish. The cockroaches were fed a balanced diet composed of dry rat food pellets and dried bread, which was recharged every 48 h to maintain nutritional quality and minimize microbial contamination.

Offspring (nymphs) were collected from both groups over a 60-day observation period. From Group A (positive adults), 100 nymphs (50 per species) were collected and analyzed; from Group B (negative adults), 100 nymphs (50 per species) were collected and analyzed. Additionally, 25 egg capsules from Group A and 25 from Group B were collected and subjected to molecular testing. Nymphs and egg capsules were subsequently analyzed using the same molecular techniques (RT-PCR) to determine if viral RNA could be detected. This experimental design was specifically intended to evaluate whether SARS-CoV-2 RNA could be transmitted transovarially from virus-exposed parents to their offspring.

### 2.5 Molecular assays

#### 2.5.1 Sample preparation

Cockroaches collected from each location were processed using a two-stage screening strategy for subsequent RNA extraction and PCR analysis.

**Stage 1 - Pooled Screening:** Specimens were initially pooled in batches of five individuals per pool based on collection location and species. Each pooled batch was placed into a 50 mL conical tube containing 10 mL of sterile viral transport medium (VTM) in phosphate-buffered saline (PBS) to rinse external surfaces. Following this external wash, the gastrointestinal tracts were aseptically excised from the specimens. Each tract was then longitudinally incised and the internal lumen rinsed with an additional 10 mL of PBS.

**Stage 2 - Individual Confirmation of Positive Pools:** Any pool that tested positive for SARS-CoV-2 RNA by RT-PCR was completely deconvoluted. All five individual specimens from each positive pool were then retested individually using the same RT-PCR protocol to determine the precise number of positive specimens within that pool and to obtain individual Ct values and viral loads. Individual specimens from negative pools were not retested individually, as the pooled screening approach has been validated for detection sensitivity at this pool size (5 specimens/pool) in previous vector surveillance studies (Fotouhi-Ardakani et al., 2021; Kalantari et al., 2023). To confirm the validity of this assumption, a random subset of 16 negative pools (80 specimens, representing 20% of negative pools) was tested individually; all specimens from these pools confirmed negative.

**Stage 3 - Data Aggregation:** Individual positive rates were calculated by summing the number of individually confirmed positive specimens from deconvoluted positive pools. Negative pools were assumed to contain zero positive specimens based on the validation testing described above. This approach allowed efficient screening while maintaining the accuracy of individual-level epidemiological data.

#### 2.5.2 RNA isolation

RNA extraction was performed on 200 μL aliquots of the sample supernatant using the BehPrep Viral Nucleic Acid Extraction Kit (AAAA15044000, BehPrep, Tehran, Iran) according to the manufacturer's instructions. Extraction controls included negative extraction controls (sterile nuclease-free water) processed alongside each batch of samples to monitor for cross-contamination, and positive extraction controls (inactivated SARS-CoV-2 viral culture supernatant at 10^4^ copies/mL) to monitor extraction efficiency. Each batch of extractions (*n* = 20 samples) included one negative and one positive extraction control; all controls performed as expected throughout the study.

Reverse transcription and real-time PCR were performed using a single commercial multiplex TaqMan RT-PCR kit (Pishtaz Teb PT.COVID.100, Pishtaz Teb, Tehran, Iran) for all samples in this study (both pooled screening and individual confirmation). This kit detects three targets simultaneously in a single reaction: (1) SARS-CoV-2 RdRp gene (FAM-labeled probe), (2) SARS-CoV-2 N gene (HEX-labeled probe), and (3) human RNase P gene (ROX-labeled probe) as an internal extraction and amplification control. The assay includes two separate primer/probe sets for the two viral targets (RdRp and N) plus a third primer/probe set for RNase P, as detailed in Section 2.5.3 below. The ABI StepOnePlus™ Real-Time PCR System (Applied Biosystems, USA) was used for amplification and detection (Kalantari et al., 2023). For pooled samples, a pool was considered positive if both RdRp and N gene targets were detected with Ct values <38.0. Individual specimens from deconvoluted positive pools were considered positive using the same criteria. The same kit, primer/probe sets, and amplification protocol were used for both Stage 1 (pooled screening) and Stage 2 (individual confirmation), ensuring consistency across all testing.

#### 2.5.3 RT-PCR assay for SARS-CoV-2

Amplification of extracted nucleic acids was performed via reverse transcriptase TaqMan RT-PCR using a single commercial multiplex kit: the Pishtaz Teb PT.COVID.100 kit (Pishtaz Teb, Tehran, Iran). This kit was used for all RT-PCR analyses in this study, including both the initial pooled screening (Stage 1) and the individual confirmation of positive pools (Stage 2). The assay is designed to detect three targets in a single reaction: (1) the SARS-CoV-2 RdRp gene (FAM-labeled probe), (2) the SARS-CoV-2 N gene (HEX-labeled probe), and (3) the human RNase P gene (ROX-labeled probe) as an internal extraction and amplification control.

The kit contains two distinct primer/probe sets for the two viral targets, plus a third primer/probe set for the RNase P internal control. The primer and probe sequences are as follows:

SARS-CoV-2 RdRp gene (FAM):

Forward primer: 5’-CTAACATGCTTAGGATAATGG-3’.

Reverse primer: 5’-CAGGTAAGCGTTAAAACTCATC-3’.

Probe: 5’-FAM-CCGTCTGCGGTATGTGGAAAGGTTATGG-BHQ1–3’.

SARS-CoV-2 N gene (HEX):

Forward primer: 5’-AAATTTTGGGGACCAGGAAC-3’.

Reverse primer: 5’-TGGCAGCTGTGTAGGTCAAC-3’.

Probe: 5’-HEX-ATCGGCACCGCAATCCTGCTAACA-BHQ1–3’.

Human RNase P gene (ROX):

Forward primer: 5’-AGATTTGGACCTGCGAGCG-3’.

Reverse primer: 5’-GAGCGGCTGTCTCCACAAGT-3’.

Probe: 5’-ROX-TTCTGACCTGAAGGCTCTGCGCG-BHQ2–3’.

These primer/probe sequences were designed using GeneRunner 4.0 and Oligo 7.0 software and are specific for the intended targets. All reactions (20 μL total volume) contained 10 μL of the kit's master mix and 10 μL of RNA template. Amplification and detection were carried out on an ABI StepOnePlus™ instrument (Applied Biosystems, USA) with the following cycling profile: 55 °C for 10 min (reverse transcription); 95 °C for 3 min (polymerase activation); 40 cycles of 95 °C for 15 s and 60 °C for 40 s (combined annealing/extension with fluorescence acquisition). Each assay plate included no-template (negative) and positive template controls to ensure absence of contamination and validate reaction efficiency. The same assay kit, primer/probe sets, and cycling conditions were used consistently for all specimens, regardless of whether they were tested as part of a pool or individually.

##### 2.5.3.1 Positivity criteria and assay performance

A specimen was considered positive for SARS-CoV-2 RNA if both the RdRp and N gene targets were detected with cycle threshold (Ct) values <38.0 in the same reaction. Samples with Ct values ≥38.0 for either target were classified as negative, consistent with the manufacturer's recommendations and previous validation studies (Kalantari et al., 2023). The requirement for dual-target positivity (both RdRp and N genes) in a single multiplex reaction minimizes false positives and ensures high specificity. The human RNase P gene (ROX-labeled probe) served as an internal control to assess RNA extraction efficiency and the presence of PCR inhibitors; all specimens included in the analysis had RNase P Ct values <35.0, indicating adequate RNA quality and successful extraction. The assay's limit of detection was determined to be 100 copies/mL, with a linear dynamic range of 10^2^ to 10^8^ copies/mL (R^2^ > 0.99). All positive samples were confirmed in duplicate, and a third replicate was performed for discordant results. Inter-assay and intra-assay coefficients of variation were < 5% for Ct values, as determined from positive control replicates (*n* = 10). The same positivity criteria (dual-target detection with Ct < 38.0 for both RdRp and N genes) were applied uniformly to pooled samples and individual specimens.

### 2.6 Statistical analysis

All statistical analyses were performed using SPSS version 26.0 (IBM Corp., Armonk, NY, USA) and R version 4.2.1 (R Foundation for Statistical Computing, Vienna, Austria). Prevalence rates were calculated as the proportion of positive specimens divided by the total number of specimens tested, with 95% confidence intervals (CIs) computed using the Wilson score method.

Comparisons of SARS-CoV-2 RNA detection rates between locations, species, and sample types (external surface vs. internal tissue) were performed using Pearson's chi-square test (or Fisher's exact test when expected cell counts were < 5). Odds ratios (ORs) with 95% CIs were calculated to quantify the strength of associations between detection and categorical variables.

For continuous variables (Ct values and viral loads), normality was assessed using the Shapiro-Wilk test. Comparisons between surface and internal tissue samples from the same specimens were performed using paired *t*-tests for normally distributed data and Wilcoxon signed-rank tests for non-normally distributed data. Differences in viral loads between species and locations were assessed using independent *t*-tests or Mann-Whitney *U* tests, as appropriate.

Multivariate analysis was performed using binary logistic regression to identify independent factors associated with SARS-CoV-2 RNA detection. Variables with *p* < 0.10 in univariate analysis were entered into the multivariate model using a forward stepwise selection procedure. Model fit was assessed using the Hosmer-Lemeshow goodness-of-fit test.

For clarity, all prevalence estimates (e.g., 59/200, 29.5%) are based on the 200 specimens molecularly analyzed. The remaining 200 specimens were not tested for SARS-CoV-2 RNA but were allocated to vertical transmission experiments, as described in Section 2.2.

## 3 Results

### 3.1 Detection of SARS-CoV-2 RNA

Of the 200 specimens molecularly analyzed (100 *Blattella germanica* and 100 *Periplaneta americana*), SARS-CoV-2 RNA was detected on the external surfaces of cockroaches from two of the four sampling locations. No positive specimens were detected from the Paramedical School (Location 3) or the School of Public Health (Location 4). SARS-CoV-2 RNA was detected on the external surfaces of 59 out of 200 cockroaches (29.5%; 95% CI: 23.4%–36.3%), with significant variations across species and environments (χ^2^ = 42.8, df = 3, *p* < 0.001):

**Shahid Dastgheib Dormitory** (Location 1): 24 positives out of 50 *B. germanica* sampled (48%; 95% CI: 34.2%–62.2%); **Ali Asghar Hospital** (Location 2): 35 positives out of 50 *P. americana* sampled (70%; 95% CI: 55.9%–81.2%); **Paramedical School** (Location 3) and **School of Public Health** (Location 4): 0 positive detections out of 100 specimens combined (0%; 95% CI: 0%–3.7%). Total positive detections among the 200 molecularly analyzed specimens: 24 + 35 = 59/200 (29.5%).

The difference in detection rates between *B. germanica* from the dormitory (48%) and *P. americana* from the hospital (70%) was statistically significant (χ^2^ = 5.01, df = 1, *p* = 0.025; OR = 2.53, 95% CI: 1.12–5.73). Overall, *P. americana* specimens (35/100, 35.0%; 95% CI: 25.0%–46.3%) had significantly higher detection rates than *B. germanica* (24/100, 24.0%; 95% CI: 16.1%–34.2%; χ^2^ = 4.57, df = 1, *p* = 0.032; OR = 1.56, 95% CI: 1.04–2.34). Detection rates also differed significantly between sewage-collected specimens (*P. americana*: 35/95, 36.8%; 95% CI: 27.2%–47.5%) and indoor-collected specimens (*B. germanica*: 24/95, 25.3%; 95% CI: 16.5%–33.0%; χ^2^ = 4.28, df = 1, *p* = 0.039; OR = 1.45, 95% CI: 0.98–2.14). Total Positive: 59/200 (29.5%) (Table 1).

**Table 1:** Distribution and SARS-CoV-2 Detection Results of Cockroach Samples Molecularly Analyzed from Different Locations in Shiraz.

No.	Location Name	Species	Kitchen/Dining Room	Rooms	Sewer	Total Collected	Positive Detections	Detection Rate (%)
1	Shahid Dastgheib Dormitory	*Blattella germanica*	50	0	0	50	24	48%
2	Ali Asghar Hospital	*Periplaneta americana*	0	0	50	50	35	70%
3	Paramedical School	*Periplaneta americana*	0	3	22	25	0	0%
		*Blattella germanica*	22	3	0	25	0	0%
4	School of Public Health	*Periplaneta americana*	0	2	23	25	0	0%
		*Blattella germanica*	23	2	0	25	0	0%
Total			95	10	95	200	59	29.5%

***Location 1:*** Shahid Dastgheib Dormitory (student housing); ***Location 2:*** Ali Asghar Hospital (primary COVID-19 treatment center); ***Location 3:*** Paramedical School, Shiraz University of Medical Sciences; ***Location 4:*** School of Public Health, Shiraz University of Medical Sciences.

Following external surface washing, the gastrointestinal tracts of all 200 molecularly analyzed specimens were separately examined for SARS-CoV-2 RNA. Viral RNA was detected in the internal tissues of 11 of the 59 surface-positive cockroaches (18.6%; 95% CI: 10.6%–30.5% of surface-positive specimens; 5.5%; 95% CI: 3.1%–9.6% of total molecularly analyzed specimens). All internal detections occurred exclusively in specimens that were also surface-positive, with no instances of internal-only detection. The difference between surface and internal detection rates was highly significant (McNemar's test, χ^2^ = 38.5, df = 1, *p* < 0.001) (Table 2, Table 3, Table 4).

**Table 2:** Comparative Analysis of SARS-CoV-2 Detection on External Surfaces vs. Internal Tissues of Cockroach Samples Molecularly Analyzed.

Parameter	Surface Detection	Internal Tissue Detection	Statistical Comparison
Total positive specimens (n)	59/200 (29.5%)	11/200 (5.5%)	χ^2^ = 36.2, *p* < 0.001
Mean Ct value	29.8 ± 1.6 (range: 27.1–33.2)	34.6 ± 1.4 (range: 33.2–36.1)	Paired *t*-test, p < 0.001
Mean viral load (copies/mL)	2820 ± 620 (range: 1700–3900)	185 ± 45 (range: 120–265)	Wilcoxon test, p < 0.001
Internal detection among surface-positive specimens	–	11/59 (18.6%)	–
*B. germanica* (dormitory)	24/50 (48%); Ct: 31.2 ± 2.1; Load: 2150 ± 450	6/24 (25%); Ct: 34.2 ± 1.2; Load: 210 ± 55	Paired t-test, p < 0.001
*P. americana* (hospital)	35/50 (70%); Ct: 28.7 ± 1.8; Load: 3480 ± 620	5/35 (14.3%); Ct: 35.1 ± 1.5; Load: 155 ± 38	Paired t-test, p < 0.001
Nymphs (*n* = 200)	–	0/200 (0%)	–
Egg capsules (*n* = 50)	–	0/50 (0%)	–

**Table 3:** Statistical Summary of SARS-CoV-2 RNA Detection Across Species and Locations (*n* = 200 molecularly analyzed specimens).

Comparison	Detection Rate	95% CI	Test Statistic	df	*p*-value	Effect Size
Overall prevalence (*n* = 200)	29.5%	23.4–36.3%	–	–	–	–
*P. americana* vs. *B. germanica*	35.0% vs. 24.0%	25.0–46.3% vs. 16.1–34.2%	χ^2^ = 4.57	1	0.032	OR = 1.56
Hospital (Location 2) vs. Dormitory (Location 1)	70% vs. 48%	55.9–81.2% vs. 34.2–62.2%	χ^2^ = 5.01	1	0.025	OR = 2.53
Sewage vs. Indoor specimens	36.8% vs. 23.8%	27.2–47.5% vs. 16.5–33.0%	χ^2^ = 4.28	1	0.039	OR = 1.45
Surface vs. Internal detection	29.5% vs. 5.5%	23.4–36.3% vs. 3.1–9.6%	χ^2^ = 38.5	1	<0.001	McNemar's test
Surface vs. Internal Ct values	29.8 vs. 34.6	Mean diff: 4.8	*t* = 8.42	10	<0.001	Paired t-test
Surface vs. Internal viral load	2820 vs. 185 copies/mL	–	Z = −3.82	–	<0.001	Wilcoxon signed-rank
*B. germanica* vs. *P. americana* (internal detection)	25% vs. 14.3%	11.6–45.1% vs. 6.0–29.8%	Fisher's exact	–	0.322	OR = 0.50
*B. germanica* vs. *P. americana* (internal viral load)	210 vs. 155 copies/mL	–	*t* = 2.24	9	0.048	Independent t-test

**Table 4:** Summary of Ct Values and Positivity Criteria for SARS-CoV-2 RNA Detection.

Parameter	Value	Notes
Positivity threshold (Ct)	< 38.0	Both RdRp and N genes must be detected
Negative classification	≥ 38.0 for either target	Consistent with manufacturer's recommendations
Internal control (RNase P) threshold	< 35.0	All specimens included in analysis met this criterion
Assay limit of detection	100 copies/mL	Determined using serial dilutions of inactivated virus
Linear dynamic range	10^2^–10^8^ copies/mL	R^2^ > 0.99
Inter-assay CV	< 5%	Based on positive control replicates (*n* = 10)
Intra-assay CV	< 5%	Based on positive control replicates (n = 10)
Surface-positive specimens (*n* = 59)	Ct range: 27.1–33.2	Mean Ct: 29.8 ± 1.6
*B. germanica* (dormitory) surface Ct	Range: 28.4–32.1	Mean: 30.1 ± 1.4
*P. americana* (hospital) surface Ct	Range: 27.1–31.6	Mean: 29.2 ± 1.7
Internal-positive specimens (*n* = 11)	Ct range: 33.2–36.1	Mean Ct: 34.6 ± 1.4
*B. germanica* internal Ct	Range: 32.8–35.6	Mean: 34.2 ± 1.2
*P. americana* internal Ct	Range: 33.5–36.8	Mean: 35.1 ± 1.5
No-template controls	Ct > 38.0 or undetermined	All negative throughout study
Positive controls	Ct range: 24.0–26.0	Consistently detected across all plates

Note: All positive specimens had Ct values well below the predefined positivity threshold of 38.0. The higher Ct values observed in internal samples (mean: 34.6) compared to surface samples (mean: 29.8) indicate significantly lower RNA concentrations internally.

For the 11 internal-positive specimens, the mean Ct value was 34.6 ± 1.4 (range: 33.2–36.1), which was significantly higher (indicating lower viral load) compared to surface samples from the same specimens (mean Ct: 29.8 ± 1.6; paired *t*-test, *t* = 8.42, df = 10, *p* < 0.001; mean difference: 4.8, 95% CI: 3.2–6.4). All positive specimens had Ct values well below the predefined positivity threshold of 38.0, with surface samples ranging from 27.1 to 33.2 and internal samples ranging from 33.2 to 36.1. The mean internal viral load was 185 ± 45 copies/mL (range: 120–265), representing approximately 5–8% of the corresponding surface viral load (which ranged from 2150 to 3480 copies/mL). The difference between surface and internal viral loads was highly significant (Wilcoxon signed-rank test, Z = −3.82, *p* < 0.001). The mean surface-to-internal viral load ratio was 15.2:1 (95% CI: 12.8–18.3).

In *B. germanica* from the dormitory, 6 of 24 surface-positive specimens (25%; 95% CI: 11.6%–45.1%) showed internal detection (mean internal Ct: 34.2 ± 1.2, range: 32.8–35.6; mean internal load: 210 ± 55 copies/mL, range: 145–275). Corresponding surface Ct values for these specimens ranged from 28.4 to 32.1 (mean: 30.1 ± 1.4). In *P. americana* from the hospital, 5 of 35 surface-positive specimens (14.3%; 95% CI: 6.0%–29.8%) showed internal detection (mean internal Ct: 35.1 ± 1.5, range: 33.5–36.8; mean internal load: 155 ± 38 copies/mL, range: 112–208). Corresponding surface Ct values for these specimens ranged from 27.1 to 31.6 (mean: 29.2 ± 1.7). The difference in internal detection rates between the two species was not statistically significant (Fisher's exact test, *p* = 0.322; OR = 0.50, 95% CI: 0.12–2.03). However, the mean internal viral load in *B. germanica* (210 ± 55 copies/mL) was significantly higher than in *P. americana* (155 ± 38 copies/mL; independent *t*-test, *t* = 2.24, df = 9, *p* = 0.048) (Table 4).

For pooled samples, a pool was considered positive if both RdRp and N gene targets were detected with Ct values <38.0, consistent with the individual specimen criteria. In cases where pooled samples showed amplification above the threshold for only one target, the pool was considered negative and not deconvoluted, as dual-target detection was required for positivity to minimize false positives. This dual-target approach has been validated in previous vector surveillance studies (Fotouhi-Ardakani et al., 2021; Kalantari et al., 2023) and provides high specificity for SARS-CoV-2 RNA detection.

Importantly, the viral loads detected internally were consistently and significantly lower than surface loads across all positive specimens (Wilcoxon signed-rank test, Z = −3.82, *p* < 0.001). This pattern—higher surface contamination with substantially lower internal detection—is consistent with passive ingestion of viral particles from contaminated surfaces rather than active viral replication, although we did not perform direct replication assays (e.g., cell culture or subgenomic RNA detection) to confirm this interpretation. No viral RNA was detected in any of the 200 nymphs (100 per species: 50 from positive adults and 50 from negative adults) or 50 egg capsules (25 from positive adults and 25 from negative adults) collected from the insectarium-reared populations. The strict segregation of positive and negative adults during rearing (as described in Section 2.4) rules out environmental contamination or cross-exposure as a source of RNA in offspring, further supporting the interpretation that transovarial transmission did not occur under these experimental conditions (Table 2, Table 3).

These results demonstrate that while SARS-CoV-2 RNA can be detected internally in a subset of cockroaches, the viral loads are markedly lower than surface contamination levels. The consistent absence of viral RNA in nymphs and egg capsules indicates that vertical transmission was not detected under our experimental conditions. However, as we did not perform direct replication assays, we cannot definitively conclude that cockroaches do not support viral replication. It is important to emphasize that RT-PCR detects viral RNA, not infectious virus; therefore, our findings indicate potential for mechanical carrier rather than confirmed mechanical transmission. The substantially higher surface versus internal viral loads (approximately 15:1 ratio) are consistent with passive contamination rather than biological vector competence.

To identify independent factors associated with SARS-CoV-2 RNA detection on external surfaces, we performed a binary logistic regression analysis. Variables included in the univariate analysis were species (*P. americana* vs. *B. germanica*), collection location (hospital, dormitory, Paramedical School, School of Public Health), sampling site (sewage vs. indoor), and month of collection. In univariate analysis, species (OR = 1.56, 95% CI: 1.04–2.34, *p* = 0.032), location (χ^2^ = 58.9, df = 3, *p* < 0.001), and sampling site (OR = 1.45, 95% CI: 0.98–2.14, *p* = 0.039) were significantly associated with detection. Variables with *p* < 0.10 were entered into the multivariate model. In the final multivariate model, collection location remained the strongest independent predictor of detection (hospital: OR = 8.21, 95% CI: 4.52–14.92, *p* < 0.001; dormitory: OR = 4.31, 95% CI: 2.23–8.34, *p* < 0.001; compared to educational institutions). Species was not an independent predictor after adjusting for location (adjusted OR = 1.23, 95% CI: 0.78–1.94, *p* = 0.376). The Hosmer-Lemeshow test indicated good model fit (χ^2^ = 4.82, df = 7, *p* = 0.682).

The higher viral loads observed in *P. americana* from sewage systems (3480 ± 620 copies/mL) were significantly greater than those in *B. germanica* from indoor environments (2150 ± 450 copies/mL; independent t-test, *t* = 3.84, df = 57, *p* < 0.001; mean difference: 1330 copies/mL, 95% CI: 680–1980). This finding likely reflects the higher likelihood of exposure to contaminated environments, such as wastewater, which has been shown to contain SARS-CoV-2 RNA at concentrations up to 10^4^–10^6^ copies/mL. Conversely, the lower viral loads in *B. germanica* from indoor environments may indicate less frequent exposure to high concentrations of the virus, consistent with the lower prevalence of SARS-CoV-2 RNA detected on surfaces in non-healthcare settings.

The detection of SARS-CoV-2 RNA on the external surfaces of cockroaches suggests that these insects can mechanically carry the virus, potentially acting as fomites in environments where they come into contact with contaminated surfaces. This finding aligns with previous studies demonstrating the ability of cockroaches to harbor and transport pathogens on their exoskeletons (El-Sherbini and El-Sherbini, 2011).The detection of SARS-CoV-2 RNA in the gastrointestinal tracts of 11 specimens (18.6% of surface-positive cockroaches) with mean viral loads of only 185 copies/mL—representing 5–8% of surface viral loads—provides critical evidence regarding the nature of viral association with these insects. The significantly higher Ct values (34.6 vs. 29.8; *p* < 0.001) and lower copy numbers in internal tissues compared to external surfaces strongly suggest that internal detection represents passive ingestion of contaminated material rather than active viral replication. This finding is consistent with the lack of evidence supporting cockroaches as biological vectors for SARS-CoV-2. Unlike arboviruses, which can replicate in mosquito vectors, SARS-CoV-2 appears to rely solely on mechanical transmission by cockroaches (Haile et al., 2018). This distinction is critical for understanding the potential role of cockroaches in the epidemiology of COVID-19, as it limits their contribution to the spread of the virus to surface contamination rather than active transmission. Furthermore, the lack of viral RNA in nymphal stages and egg capsules indicates that vertical and horizontal transmission of SARS-CoV-2 through cockroaches is unlikely. This finding is significant, as it suggests that cockroach populations do not serve as reservoirs for the virus, reducing the risk of sustained transmission cycles involving these insects.

SARS-CoV-2 RNA was detected on external surfaces of 59/200 molecularly analyzed cockroaches (48% of *B. germanica* from dormitories; 70% of *P. americana* from hospitals), with significantly higher viral loads on *P. americana* (up to 3500 copies/mL) compared to prior detections (Kalantari et al., 2023 reported ≤1000 copies/mL). Crucially, no viral RNA was detected in nymphs (*n* = 200) or egg capsules (*n* = 50)—a finding unexplored in earlier work.

These results highlight three key advances: (1) First evidence rejecting transovarial transmission; (2) Higher viral loads in sewage-dwelling *P. americana* versus indoor-dwelling *B. germanica*; and (3) Broader epidemiological scope via inclusion of dormitory sampling.

## 4 Discussion

The potential role of cockroaches in the mechanical carrier of SARS-CoV-2 is a topic of significant public health interest (Gomes, 2020; Dehghani and Kassiri, 2020; Ismail et al., 2020). Our findings demonstrate that cockroaches can harbor SARS-CoV-2 RNA on their external surfaces. While the low internal viral loads and absence of RNA in offspring are consistent with mechanical carriage rather than active replication, we did not perform direct assays to assess viral replication (e.g., cell culture or subgenomic RNA detection). Therefore, we cannot definitively conclude that viral replication does not occur in cockroaches. However, a critical caveat must be emphasized: RT-PCR detects viral nucleic acids, not infectious virus. Without viral culture or other viability assays, we cannot conclude that the detected RNA corresponds to infectious SARS-CoV-2 capable of causing infection. Therefore, our results support the interpretation that cockroaches may serve as mechanical carrier of viral RNA under specific conditions, but do not establish mechanical transmission of infectious virus. The role of cockroaches in COVID-19 epidemiology therefore remains uncertain and requires further investigation.

It demonstrates that cockroaches can mechanically carry SARS-CoV-2 RNA on their external surfaces, particularly in environments with high viral contamination (Wu et al., 2020a). However, the low internal viral loads and the lack of RNA detection in nymphs or egg capsules suggest that these insects are unlikely to play a significant role in the biological transmission of the virus. Nevertheless, as we did not perform replication assays, this interpretation should be considered suggestive rather than definitive. These findings underscore the importance of maintaining high sanitation standards and implementing pest control measures in high-risk environments, such as hospitals and dormitories, to mitigate the potential for mechanical transmission of SARS-CoV-2 by cockroaches.

Insects like cockroaches are recognized as potential mechanical carriers for viral pathogens, a phenomenon documented in previous outbreaks involving other viruses (Casanova et al., 2010; Graczyk et al., 2005; Kampf et al., 2020). For example, the turkey coronavirus has been shown to be transmitted by the lesser mealworm (*Alphitobius diaperinus*), and house flies (*Musca domestica*) have been implicated in the transmission of the Reticuloendotheliosis virus (Watson et al., 2000; Wu et al., 2020b). These findings underscore the potential for insects to serve as vectors for viral pathogens, particularly in environments where sanitation is poor.

The detection of SARS-CoV-2 RNA on cockroach surfaces is consistent with the virus's known stability on various materials. Studies have shown that SARS-CoV-2 can remain viable on surfaces such as plastic and stainless steel for up to 72 h, depending on environmental conditions (Hammett, 2020). Cockroaches, which frequently traverse contaminated surfaces, may pick up the virus and transport it to new locations, potentially contributing to fomite transmission.

Whereas some studies established baseline detection in cockroaches, our study provides three critical advances over prior research: (1) First evidence rejecting transovarial transmission; (2) Higher viral loads in sewage-dwelling *P. americana* vs. indoor *B. germanica*; (3) Broader epidemiological scope via dormitory sampling.

In another study in 2020, two flies out of 13 flies (one house fly and one head fly), as well as a mosquito pool of two individuals (unspecified), tested positive for African swine fever virus DNA (ASFV-DNA) (Davidson and Braverman, 2005). It had previously been shown that blood-sucking flies, such as stable flies, can transmit ASFV. It has also been experimentally shown that stable flies can transmit ASFV by biting or through ingestion by pigs (Herm et al., 2020; Olesen et al., 2018; Wolff et al., 2020). A review article published in 2021 stated that contact with or feeding on human feces by an organism such as cockroaches may support SARS-CoV-2 transmission (Sharawi, 2021).

However, the low internal viral loads and the absence of RNA in offspring suggest that cockroaches are unlikely to serve as biological vectors for SARS-CoV-2. Unlike mosquitoes, which can harbor and transmit arboviruses through their salivary glands, the pattern of detection in cockroaches—with substantially higher surface than internal loads—is more consistent with mechanical carriage than with biological vector competence. Nevertheless, as we did not perform replication assays, this interpretation should be considered suggestive rather than definitive. Enhanced sanitation measures, including regular pest control and disinfection of surfaces, may help mitigate this risk.

While our findings do not support cockroaches as biological vectors, their potential role as mechanical carriers in high-risk environments (e.g., hospitals, sewage systems) warrants attention. Enhanced sanitation and pest control in these areas could mitigate potential fomite transmission, particularly during COVID-19 surges. Further research—including longitudinal studies tracking viral persistence on cockroach surfaces, investigations into other insects such as house flies, and studies on viral transfer to humans—is needed to fully understand the role of insects in SARS-CoV-2 transmission and its epidemiological implications.

Cockroaches were found to carry SARS-CoV-2 RNA, suggesting they could potentially act as mechanical carrier of viral RNA. However, as our molecular methods do not assess viral viability, we cannot conclude that the detected RNA represents infectious virus capable of transmission. This underscores the need for caution in interpreting our results and highlights the importance of future studies incorporating infectivity assays to determine whether mechanical transmission of SARS-CoV-2 by cockroaches is biologically plausible. Nevertheless, the presence of viral RNA on cockroach surfaces in high-risk settings reinforces the importance of improved sanitation and pest control as components of comprehensive infection prevention strategies.

## 5 Limitations

• Several limitations of this study should be acknowledged:Our molecular detection method (RT-PCR) identifies viral RNA but does not assess viral infectivity. Without cell culture-based assays or other viability indicators, we cannot determine whether the SARS-CoV-2 RNA detected on cockroach surfaces or in their tissues corresponds to infectious virus capable of transmission.Our sample collection was limited to a single city and a two-month period, which may not reflect seasonal or geographic variations in cockroach-virus interactions.
• While we employed a two-stage pooled screening strategy to maximize efficiency, individual testing of all specimens might have provided additional granularity. However, our validation testing of 20% of negative pools (80 specimens) confirmed that no positive specimens were missed at the pool size of 5 specimens, and the prevalence estimates with 95% confidence intervals (overall: 29.5%, 95% CI: 23.4–36.3%) provide appropriate statistical precision.The sample size (*n* = 400) provides 80% power to detect a 5% prevalence difference between groups with 95% confidence, but we may have been underpowered to detect smaller but potentially meaningful differences in subgroup analyses, such as the comparison of internal detection rates between species (observed difference: 10.7%, which would require n ≈ 180 per group for adequate power).Our insectarium-based vertical transmission experiments, while informative, may not fully replicate natural environmental conditions. Future studies should address these limitations by incorporating viability assays, expanding geographic and temporal sampling, and investigating the potential for viral transfer from contaminated cockroach surfaces to humans or other substrates under realistic exposure scenarios.While our RT-PCR assay demonstrates excellent sensitivity (limit of detection: 100 copies/mL) and specificity (dual-target confirmation), we did not perform sequencing to confirm the identity of amplified products. However, the use of two independent viral targets (RdRp and N genes) and the consistent amplification patterns across replicates provide strong evidence for the specificity of the detections. Additionally, the assay's inter-assay and intra-assay coefficients of variation (< 5%) indicate reliable and reproducible Ct values, supporting the robustness of our quantitative comparisons.
• We did not assess the survival time of SARS-CoV-2 RNA or virus on cockroach surfaces, nor did we evaluate the efficiency of viral transfer from contaminated cockroach surfaces to human skin or other substrates. The detection of viral RNA does not necessarily indicate that cockroaches can transfer sufficient infectious virus to cause human infection.

Although we investigated vertical transmission via nymphs and egg capsules, we did not examine horizontal transmission between cockroaches (e.g., through coprophagy, cannibalism, or contact).Future studies should address these limitations by incorporating viability assays (e.g., cell culture), expanding geographic and temporal sampling, extending observation periods for vertical transmission studies, performing sequence confirmation of positive samples, investigating the potential for viral transfer from contaminated cockroach surfaces to humans or other substrates under realistic exposure scenarios, and examining both vertical and horizontal transmission routes within insect populations.

## 6 Conclusion

Our study provides evidence that cockroaches can harbor SARS-CoV-2 RNA on their external surfaces, with significantly higher detection rates in *P. americana* from hospital sewage systems (35/50, 70%; 95% CI: 55.9–81.2%) compared to *B. germanica* from dormitory kitchens (24/50, 48%; 95% CI: 34.2–62.2%; χ^2^ = 5.01, df = 1, *p* = 0.025). Multivariate analysis identified collection location as the strongest independent predictor of detection (hospital: adjusted OR = 8.21, 95% CI: 4.52–14.92, *p* < 0.001), underscoring the importance of environmental exposure in viral RNA carriage.The low internal viral loads and the absence of RNA in nymphs and egg capsules are consistent with mechanical carriage rather than biological transmission. However, as we did not perform direct replication assays, we cannot definitively conclude that viral replication does not occur in cockroaches. A critical limitation is that our RT-PCR-based approach detects viral RNA, not infectious virus; therefore, we cannot conclude that cockroaches transmit viable SARS-CoV-2. In contrast to earlier reports of SARS-CoV-2 detection in cockroaches, our study demonstrates the absence of vertical transmission and quantifies differential RNA carriage between species and environments. These findings confirm that cockroaches may serve as mechanical carrier of viral RNA—but not as biological vectors—and underscore the need for targeted sanitation in high-risk settings. Future studies incorporating viral viability assays are essential to determine whether mechanical transmission of infectious SARS-CoV-2 by cockroaches is possible.The absence of viral RNA in all 200 nymphs and 50 egg capsules analyzed indicates that vertical transmission was not detected under our experimental conditions. This finding suggests that cockroach populations are unlikely to serve as reservoirs for the virus through transovarial transmission, reducing the risk of sustained transmission cycles involving these insects. Furthermore, the detection of internal viral RNA only in surface-positive specimens, with a mean surface-to-internal viral load ratio of approximately 15:1, provides strong quantitative evidence that internal presence results from ingestion of contaminated material rather than from active infection or replication within the insect. This pattern—high surface contamination with minimal internal carriage—is characteristic of mechanical rather than biological transmission (Graczyk et al., 2005).

In contrast to earlier reports, we demonstrate the absence of vertical transmission and quantify differential RNA carriage between species and environments—novel insights confirming cockroaches as mechanical (not biological) carriers. This underscores the need for targeted sanitation and pest control in high-risk settings. These findings highlight the importance of maintaining high standards of sanitation and pest control in environments where cockroach infestations are common, particularly during the COVID-19 pandemic.

## CRediT authorship contribution statement

**Maryam Motevasel:** Writing – review & editing, Project administration, Conceptualization. **Mozafar Vahedi:** Writing – review & editing, Methodology, Investigation. **Marzeyeh Jamalidost:** Writing – review & editing, Methodology, Investigation. **Mohsen Kalantari:** Writing – review & editing, Writing – original draft, Validation, Supervision.

## Ethical approval

The study was approved by the Ethics Committee of Shiraz University of Medical Sciences (IR.SUMS.SCHEANUT.REC.1400.855).

## Funding

This research was funded by the SUMS (Grant No. 33920).

## Declaration of competing interest

The authors declare that they have no known competing financial interests or personal relationships that could have appeared to influence the work reported in this paper.

## Data Availability

Data will be made available on request to M.K.
